# Lessons learned from the blockade of immune checkpoints in cancer immunotherapy

**DOI:** 10.1186/s13045-018-0578-4

**Published:** 2018-02-27

**Authors:** Xiaolei Li, Changshun Shao, Yufang Shi, Weidong Han

**Affiliations:** 1 The First Affiliated Hospital of Soochow University and Jiangsu Engineering Research Center for Tumor Immunotherapy, Institutes for Translational Medicine and Suzhou Key Laboratory of Tumor Microenvironment and Pathology, Soochow University, Suzhou, Jiangsu 215123 China; 2 Department of Molecular Biology, Immunology and Bio-therapeutic, Institute of Basic Medicine, Chinese PLA General Hospital, Beijing, 100853 China

**Keywords:** Cancer immunotherapy, Immune checkpoint blockade, Tumor microenvironment, Resistance mechanism, Combination immunotherapy

## Abstract

The advent of immunotherapy, especially checkpoint inhibitor-based immunotherapy, has provided novel and powerful weapons against cancer. Because only a subset of cancer patients exhibit durable responses, further exploration of the mechanisms underlying the resistance to immunotherapy in the bulk of cancer patients is merited. Such efforts may help to identify which patients could benefit from immune checkpoint blockade. Given the existence of a great number of pathways by which cancer can escape immune surveillance, and the complexity of tumor-immune system interaction, development of various combination therapies, including those that combine with conventional therapies, would be necessary. In this review, we summarize the current understanding of the mechanisms by which resistance to checkpoint blockade immunotherapy occurs, and outline how actionable combination strategies may be derived to improve clinical outcomes for patients.

## Background

The development of cancer immunotherapy is based on the insights from cancer development, which involve increasingly accumulating mutations that provide a diverse set of antigens that the immune system can use to distinguish cancer cells from their normal counterparts. The increased understanding of the immune system and the emergence of immune modulation techniques have led to a new era in cancer therapy, and using our own biology to treat cancer is a revolutionary idea in oncology. To ensure that the immune system does not harm the host when reacting to a foreign antigen, humans have evolved immune checkpoint proteins and machineries to quickly halt an immune response. Nevertheless, in the setting of malignancy, multiple mechanisms of immune suppression may exist that prevent effective antitumor immunity [1]. New cancer therapies are based on the accumulating knowledge regarding immune regulation and immune system checkpoints.

Immunotherapies harness the immune system, both innate and adaptive, rather than the tumor itself, to attack and destroy tumors, which represent a breakthrough in the management of malignancy and allow a deeper understanding of the interplay between tumors and the immune system [2]. It has also elicited impressive therapeutic responses in some patients, but efficacy is significantly obstructed by immune suppression, tolerance, and ineffective activation. The development of immunotherapeutics for oncology, which could be divided into agents that amplify natural immune responses as well as synthetic immunotherapies designed to initiate new responses [3], has been considered the most prospective approach to treating cancers. Immune checkpoints are cell surface receptors expressed by immune cells that regulate the activation and effector functions of T lymphocytes, which are orchestrated by a set of co-stimulatory and co-inhibitory molecules. These molecules enable self-tolerance under normal physiological contexts but frequently become coopted in malignancy [4]. The best characterized are cytotoxic T-lymphocyte protein 4 (CTLA-4) and programmed cell death protein 1 pathway (PD-1/PD-L1). However, these regulatory circuits can be “hijacked” by tumors to prevent the immune system from mounting an effective antitumor response. Accordingly, immune checkpoint blockades (ICBs) have shown activity in clinical trials, and are gaining approval for an expanding array of indications, including metastatic melanoma, renal-cell carcinoma (RCC), advanced non-small-cell lung cancer (NSCLC), classic Hodgkin’s lymphoma (HL), bladder carcinoma, Merkel cell carcinoma, head and neck cancer, and more recently, solid tumors with mismatch repair-deficiency (reviewed in refs [5–7]). The unprecedented clinical success of cancer immunotherapy has given rise to a billion-dollar business. To date, out of many ongoing drug pipelines, four immunotherapeutic agents have reached clinical practice (as described below) and many more checkpoint inhibitors are expected.

However, despite the transformative potential of ICBs, upfront clinical benefits in approved indications are not universal. The prospect of broad therapeutic efficacy of ICBs across a wide range of cancer types remains elusive, such as pancreatic ductal adenocarcinoma and metastatic castration-resistant prostate cancer, which are largely resistant to checkpoint inhibitor-based immunotherapy. Consequently, there are several overriding questions: (1) why are the responses to ICBs varied so greatly among cancer patients, (2) what is the best combination therapy using ICBs, (3) how can ICB therapy coverage be extended to the majority of cancer patients who do not see control or regression of their cancer, and (4) what predictive biomarkers can be used to distinguish responsive and unresponsive cancer patients? The answers to these questions will be revealed, to some extent, with further in-depth understanding and investigative targeting of immuno-oncology mechanisms.

Current enthusiasm for ICB therapy is justified because overwhelming evidence indicates that it is effective, albeit not in all cases, where conventional therapies were not. Nevertheless, many obstacles remain before it can be made available to more cancer patients who need immune intervention. The goal of this review is to concisely review some of the recent advances in our understanding of immuno-oncology and to detail how new insights into the mechanisms that underlie cancer immune evasion might lead to development of novel and efficacious treatments. Here, with the aim of guiding future combination trials that target specific resistance mechanisms to ICB, we discuss the current understanding of mechanisms promoting resistance to ICB therapies, and outline how actionable combination strategies which target these pathways might yield better outcomes for patients. We hope that this review will be of interest to both practicing oncologists and cancer immunologists.

## Rational for checkpoints-based immunotherapy

**Fig. 1:**
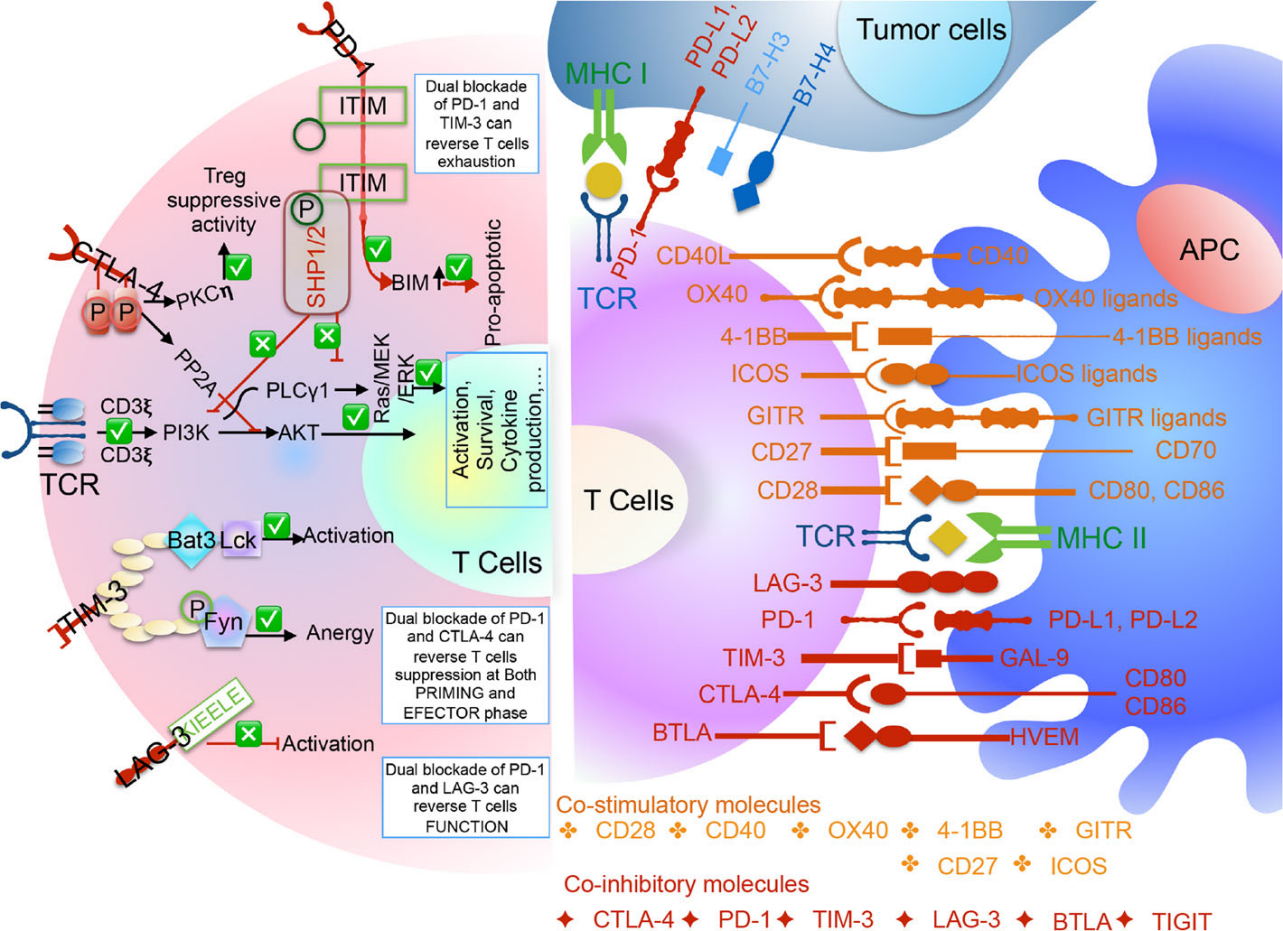
Mechanisms of action of multiple checkpoints in antitumor immunity. Co-stimulatory and co-inhibitory receptors in the immune synapse. The fine-tuning of the immune response is coordinated by a plethora of co-receptors that are responsible for amplifying or dampening the initial immune response. Most of these receptors require the TCR to specifically recognize antigens displayed by MHC molecules on APCs, to deliver their co-stimulatory or co-inhibitory signals. These interactions can take place either in secondary lymphoid sites where naïve T cells encounter antigen for the first time, or in the periphery where effector cells may be activated or suppressed. Many inhibitory receptors have ITIMs and/or ITSMs in their intracellular domains; however, some receptors have specific motifs, such as UVKM for CTLA-4 and KIEELE for LAG3. The molecular mechanisms of inhibitory receptor signaling are also illustrated and can be divided as ectodomain competition (inhibitory receptors sequester target receptors or ligands); modulation of intracellular mediators (local and transient intracellular attenuation of positive signals from activating receptors, i.e., TCR and co-stimulatory receptors); and induction of inhibitory genes. Multiple inhibitory receptors are responsible for these three mechanisms. Checkpoint therapies with antibodies to T cell inhibitory receptors (e.g., PD-1 and CTLA-4) produce durable responses in patients with many deadly malignancies. Several strategies are used to improve further the success rate of immunotherapies, including (1) combining PD-1 and CTLA-4 blockers with each other or with antagonists of other inhibitory receptors on T cells, such as TIM-3, LAG-3, TIGIT, and BTLA; (2) combining the ICB with agonists of co-stimulatory receptors of T cells, including CD27, 4-1BB, OX40, and GITR; and (3) blocking immune checkpoints in conjunction with stimulation of tumor antigen recognition using vaccines and DC activation by CD40 agonists. An alternative approach involves combining ICBs with other therapies (e.g., radiation, oncolytic viruses) that enhance tumor immunogenicity owing to ICD, and then prompt immune cells recruitment and tumor antigen presentation

Interactions between the immune system and tumor are governed by a complex network of biological pathways. Although the immune system is expected to automatically reject tumor cells as “foreign,” because of their unique and often extensive mutational profiles, the overriding outcome between the immune system and tumor is tolerance, in which tumor cells are acted as “self.” Tolerance is maintained by multiple mechanisms, including regulatory immune cells, immunosuppressive cytokines and chemokines, and so-called immune checkpoint pathways that dampen immune functions. Unopposed immune activation can be at least as damaging as an ineffective response, necessitating a dynamic system of regulatory signals to integrate the prevailing immune stimuli and direct immune responses appropriately. To evoke their proper activation, two sets of signals are required from antigen-presenting cells (APCs) regulating T cell survival, proliferation, and immune response in the lymph node [8–10]. In a normally functioning immune system, the first signal initiates via binding of T cell receptor (TCR) and a matching antigen packaged onto major histocompatibility complex (MHC) proteins on APCs. However, this interaction is not sufficient for complete T cell activation and tumor cytolysis. It is now clear that a secondary signal is needed to modulate TCR-mediated T cell activation and to promote T cell clonal expansion and cytokine secretion (Fig. 1). The best understood co-stimulatory signal pathways are engagements of CD28 on T cells with CD80 or CD86 on APCs. To ensure that T cell activation can only be stimulated by appropriate antigens and maintain their immunologic homeostasis, T cell-mediated immunity is simultaneously controlled by co-inhibitory signals. Although T cell co-stimulation was envisaged to control initial activation of naïve T cells, T cell co-stimulatory and co-inhibitory pathways have much broader immunomodulatory functions, controlling effector T (T_eff_) cells, memory T cells, and regulatory T (T_reg_) cells, as well as naïve T cells (reviewed in ref. [11]). Under physiologic conditions, a balance between co-stimulatory and co-inhibitory signals is crucial to determine whether T cells are activated or become anergic to the specific antigens displayed on the MHC molecules. These immune checkpoints are responsible for immune homeostasis and the maintenance of tolerance in normal tissue, protecting organs from unnecessary damage while immune system could still eliminate pathogens efficiently [12]. Elucidation of the complex web of co-stimulatory and co-inhibitory signals that contribute to the tug-of-war of immune regulation and their dysregulation in tumor presents clear therapeutic opportunities targeting these to enhance antitumor immunity. Tumors develop numerous strategies to avoid detection and eradication by the host immune system. An enhanced understanding of the precise activators and inhibitors of the immune system has brought about therapeutic advances in cancer treatment.

Multiple immune checkpoint pathways have been identified. The two immune-checkpoint receptors that have been most actively studied in the context of clinical cancer immunotherapy, CTLA-4 and PD-1, regulate immune responses at different levels and by different mechanisms. The clinical efficacy of antibodies that block either of these receptors implies that antitumor immunity can be enhanced at multiple levels and that combinatorial strategies can be intelligently designed, guided by mechanistic considerations and pre-clinical models. CTLA-4 expression is induced upon T cell activation and it competes with the co-stimulatory molecule CD28 for co-stimulatory ligands; in this way, CTLA-4 attenuates the early activation of naïve and memory T cells [13–15]. The use of CTLA-4 blockade to release this brake results in increased infiltration of T cells into tumors and may limit T_reg_ cell infiltration in tumor microenvironment (TME), preventing suppression of cytotoxic T cell activity by these T_reg_ cells. Although the mechanism by which CTLA-4 enhances the immunosuppressive function of T_reg_ cells remains unknown, T_reg_ cells-specific CTLA-4 knockout or blockade significantly inhibits their ability to regulate both auto-immunity and anti-tumor immunity [13].

By contrast, PD-1 is expressed on activated lymphocytes and overexpressed on exhausted lymphocytes [16]. The interaction between PD-1 and its ligands, PD-L1 and PD-L2, is a negative regulator of T cell function that serves to maintain equilibrium between T cell activation, tolerance, and immune-mediated tissue damage [5, 17]. The major stimulator of PD-L1 expression, which is primarily found on hematopoietic and epithelia cells, is mainly stimulated by IFN-γ produced by activated T cells and by natural killer (NK) cells. PD-L2 is predominantly expressed on activated dendritic cells (DCs) and macrophages, whereas PD-L1 can be expressed on many cell types, including tumor cells, immune cells, epithelial cells, and endothelial cells [5, 17]. When bound to a ligand, PD-1 lowers the threshold for apoptosis, induces anergy via blunted TCR signaling and generally leads to T cell depletion. In certain tumor cells, elevated PD-L1 expression has been observed, which leads to increased inhibition of T cell activity in favor of tumor cell survival [4]. Binding of PD-1 to tumor cells (or infiltrating immune cells)-expressed PD-L1 and APCs-expressed PD-L2 downregulates TCR signaling, resulting in reduced production of TNF-α, IFN-γ, and IL-2 [18]. In contrast to CTLA-4, PD-1 dampens the activity of T cells engaged in an ongoing immune in peripheral tissues at the time of an inflammatory response to infection and to limit autoimmunity. Similar to CTLA-4, PD-1 is also highly expressed on T_reg_ cells, where it may enhance their proliferation in the presence of ligand. Because many tumors are highly infiltrated with T_reg_ cells that probably further suppress effector immune responses, blockade of the PD-1 pathway may also enhance antitumor immune responses by diminishing the suppressive activity of T_reg_ cells [19]. The rationale for combining CTLA-4 and PD-1 blockers is strong, because although both CTLA-4 and PD-1 are expressed on T lymphocytes, these pathways have different mechanisms for inhibiting the function of these cells. Clinical testing of the combination of these two classes of ICBs showed improved clinical response in melanoma at the expense of significantly elevated frequency of toxicities [20, 21]. Combination treatments with CTLA4 and PD-1 blockers have been approved as the first line therapy for advanced melanoma patients and are being tested in other tumor types with different dose levels and intervals of anti-CTLA4 to reduce toxicity.

Investigation of these immunosuppressive interactions has led to the clinical development and licensing of new cancer treatments, which increase immune responses by using specific antibodies to block immune checkpoint molecules. Antibodies targeting CTLA-4, PD-1, and PD-L1 are currently licensed as monotherapies for various type of cancer [5, 22]. The FDA-approved ipilimumab, an antibody against CTLA-4, in 2011; two antibodies against PD-1 (pembrolizumab and nivolumab) in 2014; and an antibody against PD-L1 (atezolizumab) in 2016 [17, 23]. Aside from the clinical success of these therapies in patients with melanoma [24–27], substantial improvements could also be achieved in patients with metastatic lung cancer, kidney cancer, bladder cancer, and Hodgkin lymphoma [28–31], indicating the ground-breaking impact of immune modulation across different cancer types. Such successful clinical findings of ICBs spark hope and excitement that cancer can be efficiently treated by targeting immune cells, rather than tumors, spurring renovated interest in the immunosurveillance theory. According to this concept, tumors can only originate and progress in the context of failing immune responses, implying that one of the goals of oncotherapy should consist in reinstating the immunological control of tumor growth [32–34]. Despite significant clinical gains in the setting of treatment with ICB, limitations to this therapeutic strategy have inevitably surfaced as they have for prior generations of therapeutic strategies. Treatment with current checkpoint inhibitor monotherapy is not effective in all tumor types. On top of this, predictive biomarkers of response to ICB are currently lacking, and toxicity can be a major issue, particularly in combination strategies (reviewed in refs [35–37]). These factors, as well as an appreciation of the cost of these agents and issues with access to therapy, call for a more comprehensive understanding of the hallmarks of response to ICB to further derive more tailored strategies.

## Impediments and challenges of ICBs in cancer immunotherapy

**Fig. 2:**
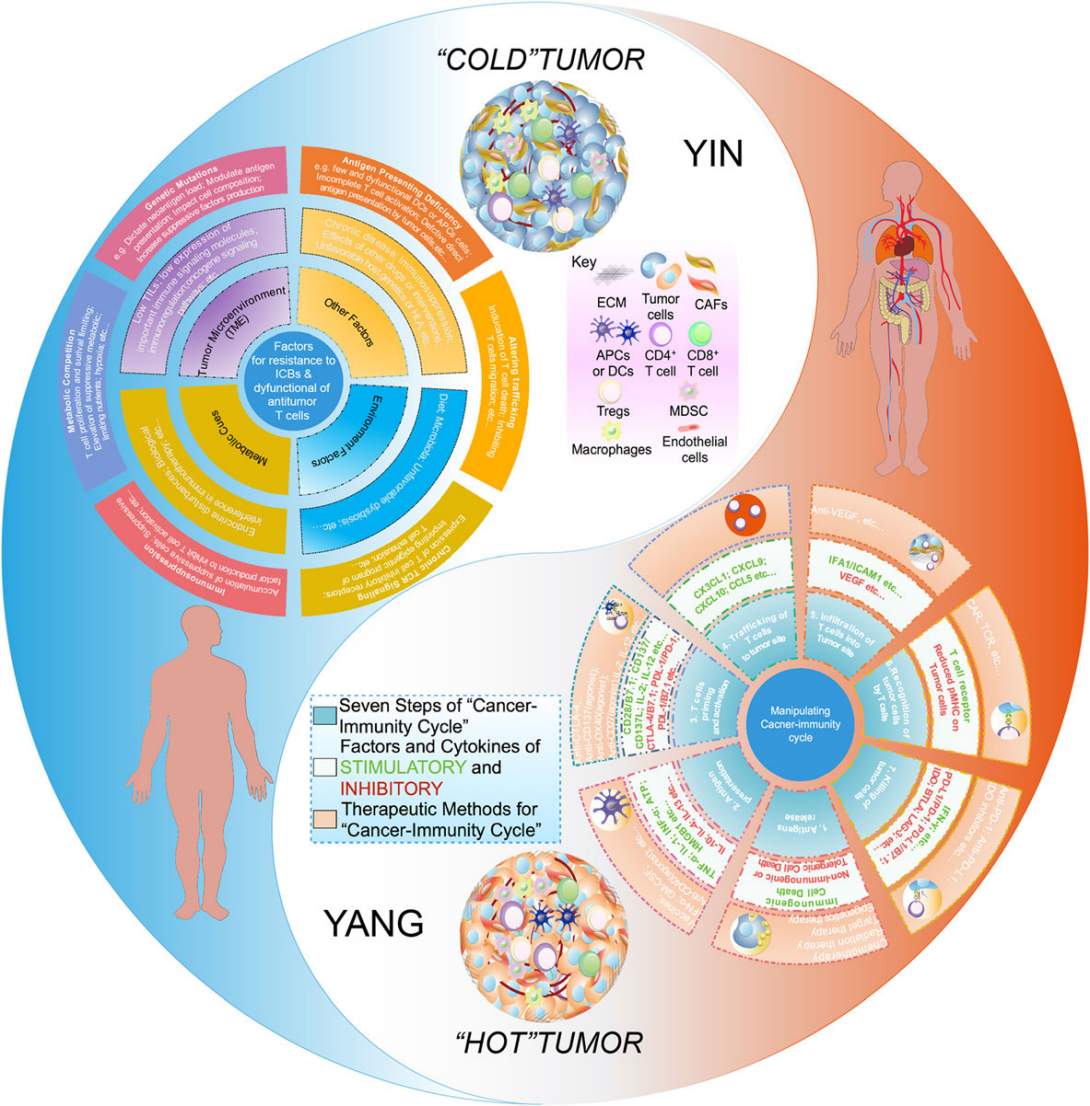
Major factors operating in the establishment of immunoresistant milieu and actionable combinations with ICBs: Yin and Yang effects. Many potential tumor, host, and environmental-related factors might explain the degree of heterogeneity seen with ICB therapy, dividing into influences from the TME, endocrine and metabolic factors, environmental factors, and other influences, i.e., age and unfavorable host genetics (Yin). Each step the cancer-immunity cycle requires the coordination of numerous factors, both stimulatory, promoting immunity and inhibitory, helping keep the process in check and reducing immune activity and/or preventing autoimmunity in nature. The numerous factors that come into play in the cancer-immunity cycle provide a wide range of potential therapeutic targets, highlighting examples of some of the therapies currently under pre-clinical or clinical evaluation. Key highlights include that vaccines can primarily promote cancer antigen presentation, anti-CTLA-4 can primarily promote priming and activation, and anti-PD-L1 or anti-PD-1 antibodies can primarily promote killing of cancer cells. Although not developed as immunotherapies, chemotherapy, radiation therapy, and targeted therapies can primarily promote release of tumor cell antigens, and inhibitors of VEGF can potentially promote T cell infiltration into tumors (Yang)

ICBs showed tremendous effects in multiple cancer types. However, responses to this form of therapy are not universal, and insights are clearly needed to identify optimal biomarkers of response and to combat mechanisms of therapeutic resistance. Great efforts are currently being undertaken to distinguish “responders” from “non-responders,” and concepts to turn the latter into the former are urgently needed. Ongoing studies indicate that both tumor-cell-extrinsic and tumor-cell-intrinsic factors contribute to the resistance (Fig. 2).

### Obstacles posed by the TME to ICB therapy

TME is composed of blood vessels, marrow-derived suppressor cells (MDSCs), APCs, lymphocytes, neutrophils, tumor-associated macrophages (TAMs) and fibroblasts, and the extracellular matrix composed of collagen and proteoglycans, and soluble factors (e.g., cytokines and growth factors), all of which may assist or hinder antitumor immune responses [38]. It is now increasingly accepted that cancer cells, rather than working alone, develop close interactions with the extracellular matrix, stromal cells, and immune cells that together form the TME, facilitating a chronic inflammatory, immunosuppressive, and proangiogenic intratumoral environment in which tumor cells could adapt and grow with a lower likelihood of detection and eradication by host immunosurveillance [38]. The importance of the immune system in protecting the body against internal threats (e.g., malignant cells) has been described as the cancer-immunity cycle [39, 40]. The cycle comprises the release of neoantigens created by oncogenesis, their release and capture by APCs for processing, antigen presentation to T cells at secondary lymphoid organs, and activation of effector cytotoxic T lymphocytes (CTLs) that then migrate and infiltrate the tumor, recognizing and killing cancer cells. One or more of the above steps required for T cell immunosurveillance are often compromised in developing tumors, leading to evasion of immune-mediate tumor control. Thus, given that the efficacy of ICB therapy is driven by T cells, this effective immune evasion can ultimately lead to failures in ICB treatment. Immune tolerance can result from suppression at any point in cancer-immunity cycle [39]. A suboptimal immune response can result from limited antigen uptake and presentation. T cells capable of responding to specific tumor antigens may be significantly reduced because of immunoediting. The ability of tumor-specific lymphocytes to be fully activated and to proliferate may be limited by a lack of effective co-stimulatory signals. Even if a robust immune response is generated, it may not last long enough to induce tumor regression. Activated T cells need to efficiently migrate to and accumulate at the tumor site, and then they also need to resist exhaustion and immunosuppression in the TME. Multiple mechanisms used by tumor cells, including alteration of the antigen presentation machinery, secretion of immunosuppressive factors that can induce apoptosis of lymphocytes, or activate negative regulatory pathways, could induce tolerance and limit the effectiveness of the immune response [41]. Tumor cells that either directly or indirectly enhance immune tolerance have a selective survival advantage thereby resulting in their outgrowth.

Given the heterogeneity in the expression levels of PD-1 ligands and their potential relevance as biomarkers for blockade of the PD-1 pathway, it is important to understand the signals that induce the expression of PD-1 ligands on tumor cells and hematopoietic cells within the TME [28, 42, 43]. Two general mechanisms for the regulation of PD-L1 by tumor cells have emerged: innate immune resistance and adaptive immune resistance. These mechanisms are not mutually exclusive and may co-exist in the same TME. Innate immune resistance refers to the constitutive expression of PD-L1 by tumor cells caused by genetic alterations or activation of certain signaling pathways. For some tumors, such as glioblastomas, it has been shown that PD-L1 expression is driven by constitutive oncogenic signaling pathways in the tumor cell. The expression on glioblastomas is enhanced upon deletion or silencing of *PTEN*, which implicates the involvement of the PI3K pathway. Similarly, constitutive ALK signaling, which is observed in certain lymphomas and occasionally in lung cancer, has been reported to drive PD-L1 expression by STAT3 signaling [44, 45]. The alternative mechanism for PD-L1 upregulation on tumors that has emerged from both clinical and pre-clinical studies reflects their adaptation to endogenous tumor-specific immune responses, known as adaptive immune resistance [46, 47]. In adaptive immune resistance, the tumor uses the natural physiology of PD-1 ligand induction that normally occurs to protect a tissue from infection-induced immune-mediated damage to protect itself from antitumor immunity. Expression of PD-L1 as an adaptive response to endogenous antitumor immunity occurs because PD-L1 is induced on most tumor cells in response to IFNs, predominantly IFN-γ. This induction also occurs in epithelial and stromal cells in normal tissues. IFN-γ is known to have dichotomous immunological properties. It can induce apoptosis of tumor cells, blood vessel disruption, and upregulation of MHC-expression on the one hand. On the other hand, IFN-γ can also promote the expression of immunosuppressive molecules such as indolaimine-2,3-deoxygenase (IDO), which inhibits immunity locally via conversion of tryptophan to kynurenines and can contribute to peripheral tolerance and can have a direct negative effect on T_eff_ cell function in coordination with upregulated PD-L1 [46, 47]. Understanding the mechanisms contributing to an effective response and resistance are of utmost importance to optimize treatment with ICBs. In this context, novel CMTM6/4 transmembrane proteins, considered as PD-L1 regulators by decreasing ubiquitination and stabilizing PD-L1, have been recently discovered in maintaining antitumor immunity [48, 49].

Resistance to ICB within TME involves components other than tumor cells, including T_reg_ cells, MDSCs, γδT cells, TAMs, and other inhibitory immune checkpoints, which may all contribute to inhibition of antitumor immune responses. Humans that lack a functional T_reg_ cell population, characterized by their expression of the *Foxp3*, develop a lethal autoimmune disorder, which can be recapitulated in mice via *Foxp3* deletion [50]. While T_reg_ cells are required to limit autoimmunity, maintain immune homeostasis, and prevent excessive tissue damage, they can be deleterious in tumor through suppression of antitumor immunity [51, 52]. Indeed, high numbers of T_reg_ cells and T_reg_ cells to T_eff_ cells ratio are considered poor prognostic factors for many tumor types, including melanoma, ovarian cancer, and colorectal carcinoma [53–55]. T_reg_ cells are known to suppress T_eff_ cell responses via secretion of certain inhibitory cytokines (e.g., IL-10, IL-35, and TGF-β) or via direct cell contact [56–60]. Multiple studies obtained from murine models have revealed that the depletion of T_reg_ cells within TME could enhance or restore antitumor immunity [61–63]. Therapeutic mAbs that target co-inhibitory receptor pathways (e.g., CTLA-4 or PD-1/PD-L1) limit T cell exhaustion, enhance CD8^+^ T cell antitumor activity, and increase T_eff_ cells to T_reg_ cells ratio in the tumors [64]. In murine models, response to CTLA-4 mAb therapy was shown to be correlated with an increase in the ratio of T_eff_ cells to T_reg_ cells [65]. This shift in the ratio of T_eff_ cells to T_reg_ cells has been found to be a result of both an increase in T_eff_ cells and depletion of T_reg_ cells in a murine tumor model, suggesting that tumors for which immunotherapy cannot increase T_eff_ cells and/or deplete T_reg_ cells to enhance the ratio of T_eff_ cells to T_reg_ cells are likely to be resistant to treatment, either initially or during the relapsed disease setting [61]. However, it is possible that tumor-infiltrating T_reg_ cells might co-exist with other immune cells, reflecting a potentially immunogenic “hot” TME. One study of patients treated with CTLA-4 mAb showed that a high baseline expression of Foxp3^+^ T_reg_ cells in the tumor was correlated with better clinical outcomes [66]. T cell exhaustion is a primary limiting factor affecting the efficacy of current cancer modalities, including CAR T cell therapies [67]. However, the promising antitumor effects noted in humans with PD-1 blockade alone offers substantial potential for reversing T cell exhaustion and improving the clinical outcome of next-generation immunotherapies [64]. Reversal of CD8^+^ T cell exhaustion and efficient control of viral load was noted following dual blockade of T_reg_ cells and PD-L1 [68], or IL-10 and PD-L1 [57], or following inhibition of TGF-β signaling [56]. Thus, there is a clear role for T_reg_ cells and its derived inhibitory cytokines in mediating T cell exhaustion, even if the precise mechanisms remain to be defined. Additional studies are ongoing to determine the impact of tumor-infiltrating T_reg_ cells on clinical outcomes for patients who receive treatment with immunotherapy agents.

MDSCs, which were initially defined in murine models, have emerged as major regulators of immune responses in various pathological conditions, including tumors. Mouse MDSCs were classified as CD11b^+^Gr-1^+^ and could be further sub-divided into the monocytic-CD11b^+^Ly6C^+^Ly6G^−^ population and the polymorphonuclear-CD11b^+^Ly6G^+^Ly6C^lo^ population [69]. Human MDSCs are classified as CD11b^+^CD33^+^HLA-DR^−^, which may co-express with other markers such as CD15, CD14, CD115, and/or CD124 [70–72]. MDSCs represent 30% of cells in the bone marrow and 2–4% cells in the spleen in normal mice. MDSCs normally differentiate into granulocytes, macrophages, or dendritic cells. However, under pathological conditions such as cancer, MDSCs become activated, rapidly expand, but remain undifferentiated. Moreover, clinical data have shown that the presence of MDSCs associates with reduced survival in several human tumors, including colorectal cancer, and breast cancer [73]. Growing evidence also suggest that heavy tumor infiltration by MDSCs correlated with poor prognosis and decreased efficacy of immunotherapies, including ICB therapy [74], adoptive T cell therapy (ACT) [75], and DCs vaccines [76]. Thus, eradicating or reprogramming MDSCs could enhance clinical responses to immunotherapy. Indeed, in multiple mouse tumor models, selective inactivation of tumor-associated myeloid cells PI3Kγ synergized with ICBs to promote tumor regression and increase survival, suggesting a critical role of suppressive myeloid cells in ICB resistance and a therapeutic potential of PI3Kγ inhibitors when combined with ICB therapy in cancer patients [77, 78]. Moreover, MDSCs have been also used to predict response to ICB [79]. Intriguingly, in 126 patients with metastatic melanoma treated with PD-1 blockade, pre-treatment MDSC numbers in the peripheral blood are correlated with response to treatment, with high MDSCs associated with reduced overall survival [80]. Analysis of peripheral blood of 59 melanoma patients treated with CTLA-4 inhibitor showed that the baseline monocytic MDSCs, neutrophils, and monocytes were more abundant in non-responders when compared to responders, which also experienced increased serum concentrations of MDSC attractants [81]. Thus, patients with existing immunosuppressive TME are poor responders to immunotherapy, and react to ICB by potentiating these immunosuppressive mechanisms. Additionally, the Fas/Fas ligand-mediated cell death pathway represents typical apoptotic signaling in many cell types, including tumor-infiltrating T cells (TILs) [82]. Tumors with apoptotic TILs, which are triggered by polymorphonuclear MDSCs in TiRP tumors, which express high level of Fas-ligand, resist immunotherapy based on ICB, cancer vaccines, or ACT [83]. Apoptosis of TILs can be prevented by interrupting the Fas/Fas-ligand axis, which enhances the antitumor efficacy of ACT in TiRP tumors, and increases the efficacy of ICB in transplanted tumors [83]. Thus, TILs apoptosis is a relevant mechanism of immunotherapy resistance, which could be blocked by interfering with the Fas/Fas-ligand axis.

γδT cells, which are innate-like T lymphocytes characterized by TCRs composed of γ and δ chains, are widely distributed in the peripheral blood and mucosal tissues. γδT cells are also a conserved population of innate lymphocytes with diverse structural and functional heterogeneity, possessing multi-functional capacities in the repair of host tissue pathogen clearance, and tumor surveillance [84]. γδT cells are important for immunosurveillance by exerting direct cytotoxicity, strong cytokine production, and indirect antitumor immune responses [85]. However, accumulating evidence suggests that certain γδT cell subsets unexpectedly drive tumor development and progression by (i) inducing an immunosuppressive TME and angiogenesis via cytokine production, (ii) interfering with DC effector function, and (iii) inhibiting antitumor adaptive T cell immunity via the PD-1/PD-L1 pathway (reviewed in ref. [86]). For example, certain γδT cell subsets also contribute to tumor progression by facilitating tumor-related inflammation and immunosuppression, with suppressive γδT cells producing IL-10 and TGF-β. TGF-β plays important roles in angiogenesis and immunosuppression by stimulating T_reg_ cells [87]. Furthermore, CD39^+^ γδT_reg_ cells are the predominant T_reg_ cells in human colorectal cancer, which have more potent immunosuppressive activity than CD4^+^ or CD8^+^ T_reg_ cells through the adenosine-mediated pathway but independent of TGF-β or IL-10. CD39^+^ γδT_reg_ cells also secrete cytokines including IL17A and GM-CSF, which may attract MDSCs, thus establishing an immunosuppressive network [88]. Additionally, an indirect regulatory role of γδT cells has been reported in colorectal cancer, whereby activated γδT17 cells in the TME also secreted other cytokines including IL-8, TNF-α, and GM-CSF, which might help support immunosuppressive MDSC [89]. Many of the immunosuppressive subsets, including γδT cells, can express inhibitory ligands, such as PD-L1, which interferes with the antitumor activity of T cells expressing the PD-1 receptor. Blockade of PD-L1 in γδT cells could enhance CD4^+^ and CD8^+^ T cell infiltration and immunogenicity in pancreatic ductal adenocarcinoma (PDA), suggesting γδT cells as central regulators of T_eff_ cells activation in cancer via novel crosstalk [90]. γδT cells are not APCs and thus not likely to present antigen to T cells, suggesting inhibition by PD-L1 expression on γδT cells and potentially other TME cells is occurring in *trans*.

TME contributes to T cell suppression via both direct contact and secretion of soluble factors. Stromal cells can limit T cell trafficking within the TME, promote T_reg_ cell development, and inhibit T cell proliferation [91]. Macrophages can be classified as pro-inflammatory and anti-inflammatory, also known as classic (M1) and alternative (M2). TAMs are another subset of cells that seem to affect responses to immunotherapy and are key coordinators of tumor-promoting angiogenesis, fibrous stroma deposition, and metastasis formation [92, 93]. Skewing or depleting TAMs could therefore affect multiple critical steps in oncogenesis and abrogate different modes of immune resistance. Different TAMs could be distinguished based on the differential expression of transcription factors, surface molecules, and the disparities in their cytokine profile and metabolism [94]. TAMs display an alternatively activated M2 phenotype known to be critical in controlling tissue homeostasis and wound healing; however, in the tumor, this phenotype is undesirable since it enables potent T cell inhibition via cytokines (e.g., IL-10), depletion of key metabolites (expression of arginase, IDO), or by contact inhibition (e.g., via PD-L1) [95]. Clinical studies have demonstrated an association between higher frequencies of TAMs and poor prognosis in human cancers [96]. Previous results suggested that macrophages could directly suppress T cell responses via PD-L1 in hepatocellular carcinoma [97], and B7-H4 in ovarian carcinoma [98]. The M2 phenotype is also critical in determining ICB efficacy as an innate wound healing and immune-suppressive gene signature was found to optimally predict non-responders prior to PD-1 mAb treatment. New findings reveal that TAMs are also important when targeting the PD-1/PD-L1 axis. Pittet and colleagues show that TAMs can capture PD-1 targeting antibodies on the T cell surface thereby considerably limiting the duration of drug efficacy [99], whereas in another paper, Weissman and collaborators reveal that TAMs also express PD-1 on their surface, which impairs their phagocytic activity [100]. To overcome the potential resistance mechanisms of macrophages, blockade of CSF1R, a receptor of macrophage-colony stimulating growth factor, in a murine model of pancreatic cancer showed decreased frequencies of TAMs, with subsequent increase in IFN production and restrained tumor progression. Tellingly, neither PD-1 nor CTLA-4 blockade could significantly reduce tumor growth in the murine model, which was similar to findings from single-agent studies in patients with pancreatic cancer [101]. However, CSF1R blockade in combination with either an antibody against PD-1 or CTLA-4, except for gemcitabine, led to improved tumor regression [101], suggesting that CSF1R blockade induced reduction of TAMs and enabled response to ICB.

### Tumor-cell-intrinsic barriers of ICB therapy

Tumor-cell-intrinsic factors that contribute to cancer immunotherapy resistance include expression or repression of certain genes and pathways in tumor cells that compromise the function of TILs in TME. Constitutive WNT signaling via the stabilization of β-catenin was shown to be associated with T cell exclusion in melanoma [102]. Active β-catenin signaling in melanoma has been previously reported to correlate with more aggressive disease [103]. The role of β-catenin signaling as an immune escape mechanism was demonstrated in genetically engineered mice developing autochthonous melanoma [103]. As in humans, activation of the oncogenic WNT-β-catenin signaling pathway in melanoma cells correlates with the absence of T cells and reduced infiltration of a subset of DCs, known as CD103^+^ DCs, due to decreased expression of CCL4 that is responsible for DCs recruitment into the TME. Thus, lack of DCs limited tumor-specific T cell priming, leading to development of resistance to PD-L1 and CTLA-4 blockers-based therapies in experimental murine tumor models [102]. Since the WNT/β-catenin oncogenic pathway has been found activated in several tumor types, this mechanism of resistance might apply to other tumors.

Oncogenic signaling pathways, such as the PI3K pathway, have been proved to associate with primary resistance to PD-1/PD-L1 blockers as well. Signaling via the PI3K-AKT-mTOR pathway contributes to tumorigenesis by impacts on a multitude of cellular processes. This pathway is commonly activated through loss of expression of the tumor suppressor PTEN, a lipid phosphatase suppressing the activity of PI3K signaling [104], which is a common phenomenon across several cancers, including 30% of melanomas, and has been found to be correlated with resistance to ICB therapy [105]. PTEN loss in melanomas is associated with significantly decreased gene expression of IFN-γ and granzyme B, with reduced infiltration of CD8^+^ T cells, and inferior outcomes after anti-PD-1 therapy. More importantly, the frequency of PTEN deletions and mutations was higher in non-T cell-inflamed tumors as compared to T cell-inflamed tumors. In murine models, the effectiveness of either anti-PD-1 or anti-CTLA4 antibodies is enhanced by treatment with a selective PI3Kβ inhibitor [105]. Similarly, oncogenic signaling through the MAPK pathway results in the production of VEGF and IL-8, among many other secreted proteins, which have known inhibitory effects on T cell recruitment and function. Combination of targeting the MAPK pathway by selective BRAF and MEK inhibitors with immunotherapy is proposed to improve the long-term outcomes of patients. More importantly, additional PI3K inhibition could be an option for BRAF plus MEK inhibitor resistant patients that receive targeted therapy in combination with ICBs [106]. Oncogenic signaling pathways are so common in many other tumors where many studies are exploring the clinical benefit of ICBs that such researches may shed some lights on additional strategies to enhance the efficacy of ICBs.

Type I and type II IFNs responses play predominant roles during distinct phases of antitumor immunity. In tumors, secretion of the type I IFNs (IFN-α and IFN-β) facilitates DC maturation that is necessary for the generation of T_eff_ cells, which return to the tumor to secrete the type II IFN (IFN-γ) to cause vascular destruction and to sensitize tumors to CTLs. TMEs with a significant lack of type I IFN-producing DCs will naturally result in limited antitumor T cell priming and thus a limited pool of useful T cells for ICB therapy to reactivate. Moreover, type I IFN activation allowed for prolonged survival when the PD-1/PD-L1 axis was subsequently targeted. Tumor cells could escape the effects of type II IFN (IFN-γ) by downregulating or mutating molecules involved in the IFNs signaling pathway, which goes by the IFN receptor chains Jak1/Jak2 and STATs [107]. Jak1/2 are tyrosine kinases essential for intracellular signaling in response to IFNs. IFN-γ released by TILs induces the expression of several IFN-stimulated genes, eventually leading to direct tumor growth arrest and apoptosis, as well as increased antigen processing and presentation, production of chemokines that attract T cells, and upregulation of PD-L1 [108]. As direct consequence of loss-of-function in Jak1/2, tumors in these patients were completely devoid of T cell infiltrates. Tumors carrying homozygous loss-of-function mutations in Jak1/2 were resistant to anti-PD-1 treatment, despite the presence of a high mutational burden [109]. Thus, Jak1/2 loss-of-function could be incorporated in the genetic screening of candidates that can be subjected to ICB therapy. Additionally, loss of JAK1 and JAK2 expression might also derive from epigenetic silencing, as it has been described in prostate cancer cell lines [110]. On the same line, genetic defects in the IFN-γ pathway have been shown to reduce the chance of response to antibodies targeting CTLA-4 in melanoma patients [111]. Analysis of tumors in patients who did not respond to therapy with the CTLA-4 blockade revealed an enriched frequency of mutations in IFNGR1 and IFNGR2, JAK2, and interferon regulatory factor 1 (IRF1). Any of these mutations would prevent signaling in response to IFN-γ and give an advantage to the tumor cells in escaping from T cell attack, thereby resulting in primary resistance to anti-CTLA-4 therapy. Loss of antigen display by tumor cells leading to acquired resistance to cancer immunotherapy may be due to mutations in the antigen-processing machinery or proteins involved in antigen presentation can lack of recognition by CD8^+^ T cells following immunotherapy [112, 113]. Such mutations were recently detected in patients who relapsed following anti-PD-1 therapy. While several other signatures might stem from the ongoing genomic, methylomic, and transcriptomic analyses in pre-existing samples from candidates to ICB, it is rather clear that high mutational burden and high CD8^+^ T cell infiltrate do not necessarily predict sensitivity to ICBs.

Tumors are a major disturbance to tissue homeostasis, creating metabolically demanding environments that encroach on the stroma and infiltrating immune cells. The unrestrained cell growth seen in cancer is often supported by aerobic glycolysis, the same metabolic pathway needed to fuel optimal effector functions in many immune cells [114]. Altered nutrient availability in tumors affects metabolic reprogramming of T cells, resulting in impaired effector functions and differentiation toward suppressive phenotypes. TILs are exposed to low extracellular glucose and glutamine due to high nutrient uptake by tumor cells [115]. Importantly, glucose is a critical substrate for the antitumor functions of T_eff_ cells and M1 macrophages, which both require engagement of aerobic glycolysis for their activation and full effector functions [116, 117]. Augmented aerobic glycolysis in tumor cells and endothelial cells places immune cells and their neighbors at odds. Glucose deprivation represses Ca2^+^ signaling, IFN-γ production, cytotoxicity, and motility in T cells and pro-inflammatory functions in macrophages [118–120]. Cytosolic Ca2^+^ concentration serves as a metabolic threshold, allowing activation of the family of transcription factors collectively named nuclear factor of activated T cells (NFAT) [121]. Consequently, glucose deprivation results in a dose-dependent decrease in IFN-γ, mediated at the translational level by decreased mTOR activity. Recently, several studies have shown that the glycolytic activities of tumor cells may restrict glucose utilization by TILs, thereby impairing antitumor immunity [119, 121]. Amino acid deprivation in the TME serves as another metabolic checkpoint regulating antitumor immunity. Glutaminolysis in tumor cells is critical to replenish metabolites by anaplerotic reactions [122], which could result in competition between tumor cells and TILs for glutamine that controls mTOR activation in T cells and macrophages. Glutamine is also a key substrate for protein *O*-GlcNAcylation and synthesis of S-2HG that regulate T_eff_ cell function and differentiation [123]. TAMs, MDSCs, and DCs could suppress TILs via expression of essential amino acid-degrading enzymes (i.e., ARG1 and IDO) [124, 125]. Indeed, inhibitors of ARG1 and IDO are under investigation as therapeutic agents in clinical trials [126]. Bioactive lipids, modified lipoproteins, and cholesterol metabolism within the tumor are also important mediators of immune cell function. DCs in the tumor can accumulate oxidized lipoproteins through scavenger receptor-mediated internalization and formation of lipid droplets, which can ultimately impair their ability to cross-present tumor antigens and activate T cells [127]. As TILs adapt to the tumor microenvironment, they progressively lose their ability to respond to TCR stimuli, produce effector cytokines, and proliferate—a process termed functional exhaustion or hyporesponsiveness. This is in part due to the upregulation of several inhibitory receptors like PD-1, LAG3, TIGIT, and CTLA-4 that desensitize T cells to tumor antigens [128]. Intriguingly, both chronic exposure to antigen and environmental triggers (i.e., glucose deprivation) could upregulate PD-1 [118, 128], which not only suppresses TCR, PI3K, and mTOR signaling in T cells but also dampens glycolysis and promotes fatty acid oxidation-features that may enhance the accumulation of suppressive T_reg_ cells in tumors [129, 130]. Extracellular adenosine, a by-product of altered tumor metabolism, induces expression of both CTLA-4 and PD-1 on T cells. Indeed, blockade of PD-1 re-energizes anabolic metabolism and glycolysis in exhausted T cells in an mTORC1-dependent manner [119, 131], breathing caution into the types of drug combinations one may consider with PD-1/PD-L1 blockades or other forms of immunotherapy. Metabolic interventions, such as the use of mTOR inhibitors, must be targeted specifically to avoid unintended intervention of immune cell function. Signaling through PD-L1 also has direct metabolic effects on cancer cells. In response to PD-L1 blockade, glucose uptake and lactate extrusion are decreased, suggesting that pathological expression of PD-L1 by cancer cells not only impairs T cell metabolism but also benefits cancer cell metabolism.

## Novel therapeutic modalities for improving the coverage of ICBs

The wide range of diverse treatment modalities for cancer has enabled us to fight the disease from many different angles; however, tumor relapse and resistance to therapy, especially in advanced-stage disease settings, remain formidable problems. The ultimate aim with ICB therapy is long-term disease control in patients with advanced malignancy. The mechanisms that underlie cancer immunotherapy differ considerably from those of other approaches to cancer treatment. Unlike chemotherapy or oncogene-targeted therapies, cancer immunotherapy relies on promoting an antitumor response that is dynamic and not limited to targeting a single oncogenic derangement or other autonomous feature of tumor cells. Cancer immunotherapy can thus lead to antitumor activity that simultaneously targets many of the abnormalities that differentiate cancer cells and tumors from normal cells and tissues. While this is unlikely to be attained by utilizing a single ICB therapy alone, it may be achieved through appropriate combinations of different therapeutics. On the basis of deeper insights gained [26, 132, 133], we will ultimately be able to refine strategies to monitor and enhance responses to ICB. Importantly, the insights gained from current basic and clinical studies using ICBs will have direct relevance to other form of therapies [134], including conventional cytotoxic chemotherapy, radiation therapy, targeted therapy, epigenetic drugs therapy, and traditional immune therapy as well as the use of other checkpoint blockade agents, and also involving tactics to either enhance endogenous T cell function or to adoptively transfer antigen-specific T lymphocytes (Fig. 2).

### Checkpoint blockade combinations

Though some of actionable approaches to combat cancer involve treatment with drugs as monotherapy, including mAbs, the majority of contemporary approaches focus on combination strategies in an effort to overcome therapeutic resistance to immunotherapy associated with treatment with single-pronged efforts [135]. A subset of patients with advance malignancy can respond to single-agent ICB, but most patients do not respond to such single-agent therapy. Thus, predictive biomarkers may provide a means to identify which patients will respond to monotherapy [35, 37, 136]. Numerous additional co-inhibitory molecules on the T cell surface have been characterized and shown to contribute to T cell exhaustion, thus, it could be beneficial or even necessary to target multiple inhibitory molecules at the same time to attempt reversal of exhaustion [128]. Combining immunological agents may increase response rates and prolong the duration of response by stimulating an antitumor immunological memory. A prime example of enhanced efficacy with combination therapy is the use of antibodies that block two key immune checkpoints, CTLA-4 and PD-1, which results in significantly higher response rates to therapy and improved clinical outcomes as compared to monotherapy, and this combination was recently FDA-approved for patients with metastatic melanoma [24, 25, 27, 137].

Building on successes of the PD-1/PD-L1 blockade or CTLA-4 blockade, numerous clinical trials of immunotherapy combinations are in progress. There is a strong rationale in combining CTLA-4 and PD-1 blockers, because despite the facts that both CTLA-4 and PD-1 are expressed on T lymphocytes, these pathways have different mechanisms for inhibiting T cell function. Thus, the superiority of combination therapy is most likely a consequence of the non-redundant functions of CTLA-4 and PD-1 as negative regulators of T cells [21, 138]. CTLA-4 expression is induced upon activation of T cells and it competes with the co-stimulatory molecule CD28 for CD80/CD86 ligands and therapy blocks the CD28 signal that is necessary for robust T cell activation and effector function, attenuating the early activation of naïve and memory T cells [16]. Despite the conventional wisdom that CTLA-4 acts early in T cell activation in secondary lymphoid tissues whereas PD-1 inhibits execution of T_eff_ cell responses in tissue and tumors, this distinction is not absolute. Beyond its role in dampening activation of T_eff_ cells, CTLA-4 plays a major role in driving the suppressive function of T_reg_ cells [13, 14]. Thus, the use of CTLA-4 blockade may affect intratumoral immune responses by enhancing T_eff_ cells function and/or depleting tumor-infiltrating T_reg_ cells [14]. Recent evidence has revealed antitumor effects of CTLA-4 blockade even when lymphocyte egress from lymph nodes was blocked by S1P inhibitors [139], indicating that this checkpoint exerts at least some effects directly in the TME beyond its function in secondary lymphoid tissues. PD-1 inhibits T cells at the effectors stage when they are present within the tissues, and its expression has been associated with T cell exhaustion [128]. Especially, PD-1 engagement limits the initial “burst size” of T cells upon antigen exposure and can partially convert T cell tolerance induction to effector differentiation [140]. CTLA-4 blocker seems to drive T cells into tumors, resulting in increased accumulation of TILs and a concomitant increase in IFN-γ production. This, in turn, can induce expression of PD-L1 in the TME, with subsequent inhibition of antitumor T cell responses, and may benefit from PD-1/PD-L1 blockade monotherapies [4]. Thus, combination treatment with CTLA-4 and PD-1 pathway blockers should enable the creation of an immunogenic TME with subsequent clinical benefit for patients regardless of the quantity of TILs or expression PD-L1 in pretreatment tumor tissues. Indeed, each of these checkpoint inhibitors has been shown to have both overlapping and unique effects on tumor-specific T cells and facilitate the conversion of a TME from “cold” to “hot.” Substantial data already exist to indicate that certain combination therapies may overcome the limitations of CTLA-4 blockade and PD-1/PD-L1 blockade monotherapies.

Besides CTLA-4 and PD-1, TILs express a diverse array of additional inhibitory co-receptors that functions as immune-checkpoint regulators, and can be targeted to boost antitumor immunity. Associated with the phenotype of some severely exhausted T cells is overexpression of multiple inhibitory molecules including TIM3, LAG3, BTLA, and TIGIT [128]. Recently, Thommen et al. showed that co-expression of PD-1, TIM3, LAG3, CTLA4, and BTLA was correlated with resistance to anti-PD-1 therapy in NSCLC. Analysis of the phenotypical and functional evolution of CD8^+^ TILs from 32 patients with NSCLC revealed that the accumulative expression of PD-1, TIM3, CTLA-4, LAG-3, and BTLA on CD8^+^ T cells was associated with tumor stage and nodal status [141]. CD8^+^ T cells expressing all the five receptors exhibited severe defects in cytokine production, proliferation, and migration. Both LAG3 and TIM3 are frequently co-expressed with PD-1 on anergic or exhausted tumor-specific CD8^+^ T cell, and for this reason, escape from PD-1 pathway blockade could be achieved by additive expression of co-inhibitory receptors on CD8^+^ T cells. Evidence for synergistic immunosuppression mediated by LAG3 and PD-1 comes from studies using double-knockout mice. Although neither *LAG3* nor *PD*-*1* single knockout animals succumb to autoimmunity, ablation of both results in multi-organ lymphocytic infiltration, and early death [142], reinforcing the notion that LAG3 and PD-1 may compensate each other in regulating T cell function. A role of dual blockade of LAG3 and PD-1 in tumor immunity is suggested by studies in which most tumors implanted in LAG3/PD-1 double-knockout mice were rejected, whereas PD-1 single-knockout mice showed delayed tumor growth. Similarly, combination immune therapy comprising anti-PD-L1/PD-1 antibodies and blocking antibodies to LAG3, TIM3, or TIGIT resulted in enhanced antitumor responses compared with single agents in pre-clinical models. Intriguingly, blocking LAG3, TIM3, or TIGIT alone had a minimal impact on tumor growth inhibition, but was active only in combination with anti-PD-L1/PD-1 treatment, suggesting that the suppressive capacity of the PD-1/PD-L1 axis is dominant over other known co-inhibitory receptors. An anti-LAG3 blocking mAb has recently entered a phase I trial (NCT01968109) that includes cohorts receiving anti-LAG3 monotherapy or combination therapy with anti-PD-1 (reviewed in ref. [143]). Another study also demonstrates that up-regulation of TIM3 on PD-1-positive lymphocytes is correlated with resistance to anti-PD-1 therapy in two fully immunocompetent mouse models of lung adenocarcinoma, and TIM3 antibody addition overcomes resistance to PD-1 blockade [144]. Hence, combination of PD-1/PD-L1 checkpoint inhibitors and blocking antibodies to co-expressed inhibitory receptors may hopefully enhance antitumor responses in patients with severely exhausted TILs.

Components of the innate immune system, such as NK cells, are now known to mediate infectious and antitumor immunity [145]. For example, many transplanted tumor cells are rejected in an NK cell-dependent manner [146]. Like activated CD8^+^ T cells, NK cells mediate target cell apoptosis via secretion of perforated granules containing perforin and granzymes. However, unlike CD8^+^ T cells, NK cells cannot recognize unique peptides in the context of classical MHC-I molecules. Instead, the molecule basis for the recognition and elimination of tumor cells by NK cells are controlled by the complex interplay of a series of activating receptors. NK cells express killer inhibitory receptors (KIRs), which inhibit the cytotoxic activity of NK cells after interaction with MHC-I on tumor cells. The importance of NK cells in murine models of cancer immunotherapy has been shown by multiple studies but is best illustrated by studies in which NK cell can fairly eradicate advanced tumors in the absence of CD8^+^ T cells, following activation with IL-15 [147]. Co-blockade of PD-1 or CTLA-4 and KIR might be beneficial due to the activation of both T- and NK cells. To that end, a fully human anti-KIR mAb has entered clinical testing. This antibody binds to the human KIR molecules KIR2DL-1, KIR2DL-2, and KIR2DL-3 as well as to KIR2DS-1 and KIR2DA-2, preventing their binding to HLA-C MHC I molecules [148]. A phase I trial of anti-KIR in acute myelogenous leukemia has been completed. Other combinations are clinical trials in which lirilumab is being combined with anti-PD-1 (nivolumab, clinical trial NCT01714739) or with anti-CTLA-4 (ipilimumab, clinical trial NCT01750580) in patients with advanced solid tumors. These trials are important in that each seeks to combine innate immune activation via anti-KIR with activation of the adaptive immune system, therefore holding the potential for additive or synergistic antitumor efficacy. Combination therapies with novel immune checkpoints might have a more favorable safety profile than the anti-PD-1 and anti-CTLA-4 combination, and clinical assessment of these approaches is needed.

Although the combination of ICBs may enhance antitumor immunity, it may also lead to an increase in the magnitude, frequency, and onset of side effects and toxicities, compared with prior experience with either antibody alone. These side effects resemble autoimmune diseases, such as dermatitis, inflammatory colitis, hepatitis, hypophysitis, and thyroiditis and, although they can usually be managed with administration of treatment involving immunosuppression, they clearly identify a requirement for careful dose titrations to define windows of clinical efficacy [24, 25, 27, 149]. Although a few combination trials included sequential treatment arms, the phased approach, in which a second agent is added on, has yet to be fully evaluated in the clinic. However, the administration of a second more toxic agent is probably not warranted in patients who will develop clinical responses to monotherapy with a less toxic agent [150]. Finally, it is not readily apparent at this time how particular combinations might be chosen for a particular patient. It is possible that certain tumor types may induce tolerance through distinct combinations of checkpoints. However, as suggested by recent data in colorectal cancer, even within a tumor type there is a large variation in which different checkpoints are expressed by a given patient [151]. If that turns out to be the case, then a “personalized” approach to combination immunotherapy might be optimal; in that scenario, tumor biopsies would be interrogated for a series of addressable checkpoints, then a personalized checkpoint blockade cocktail administered. Regardless of the eventual clinical approach, it seems likely that combined immune checkpoint blockade could be important in achieving durable responses in many, if not most, tumor types.

### Combinations ICBs with conventional therapy

Conventional treatment regimens, such as chemotherapy, radiotherapy, targeted therapy, and ADCC (occasionally mediated by tumor-targeting antibodies), can promote immunogenic cell death (ICD) of tumor cells, allow the release of tumor antigens for presentation, and thus in theory, prime the immune system. Additionally, conventional antitumor therapies can deplete immunosuppressive cells such as T_reg_ cells and MDSCs to enhance a latent antitumor immune response, thereby providing rationale behind ICB combination [152]. Patients whose tumors are “hot” would be treated with ICB therapy to elicit durable clinical benefit; however, patients whose tumors are “cold” would receive combination therapies designed to create a “hot” TME that could respond to treatment with subsequent durable clinical benefit.

Historically, immunotherapy might be most effective in cases in which there is a small burden of tumor; this reasoning also appears to be true for newer agents. Thus, combining chemotherapy with ICBs may be advantageous because chemotherapy can reduce tumor burden, potentiate antitumor response by exposing neoantigens via necrosis of the tumor, and also directly affect the tumor stromal cells. The choice of chemotherapeutic agent and timing of combinations chemotherapy with ICBs will be important, because many cytotoxic chemotherapeutics target rapidly dividing cells. The effects of chemotherapy have always been considered as being inevitably harmful to immune mechanisms; however, it is now known that these effects are rather drug-, dose-, and/or schedule-dependent. Conventional chemotherapies have been shown to cause the release of antigens and “danger signals,” also known as DAMPs, thus triggering ICD. ICD results from ordered activation of stress-response pathways correlated with the emission of danger signals by dying tumor cells, called DAMPs, which promote recognition of dying tumor cells by the innate and adaptive immune system, ultimately eliciting tumor-targeting immune responses [153]. Aside from direct cytotoxic effects on tumor cells, some chemotherapeutic agents could induce ICD and activate antitumor immune response through other possible mechanisms: DCs activation and expression of co-stimulatory molecules; enhancement of cross-priming of CD8^+^ T cells; downregulation of MDSCs and T_reg_ cells activity; promotion of tumor cell death through lytic receptors or pathways; increase in serum inflammatory cytokines and pro-inflammatory changes in TME [153, 154].

There are intriguing data that support the hypothesis that cytotoxic chemotherapy alters the immunosuppressive TME. In pre-clinical adoptive T cell and vaccine models, cyclophosphamide has been used to deplete T_reg_ cells and may augment immunotherapies in patients [155]. Additionally, various chemotherapies (i.e., gemcitabine, 5-fluorouracil, and taxanes) can cause a decrease in MDSCs [156–158]. Promising pre-clinical studies have shown that some chemotherapeutic agents could indeed sensitize tumors to ICB by promoting T cell activation and infiltration into the TME. Moreover, chemotherapy, synergized with vaccination in a pre-clinical tumor model, can enhance response to T cell-based immune therapies by sensitizing the tumor cells to T cell-induced death rather than by ICD [159]. Substantial data support the hypothesis that some chemotherapy regimens may function as a vaccine, killing tumor cells and increasing the amount of tumor-antigen processed and presented to T cells [160]. These studies provide a rationale for the exploration of chemotherapy in combination with antibodies targeting co-stimulatory and co-inhibitory receptors. With respect to the clinical application, chemotherapy using dacarbazine combined with anti-CTLA-4 was first tested in patients with metastatic melanoma. A phase II study showed that more patients responded to dacarbazine plus anti-CTLA-4 when compared to anti-CTLA-4 alone [161]. Additionally, a phase III study revealed that this combination slightly increased the over survival, when compared to dacarbazine alone. An important phase II trial in patients with stage IIIb/IV NSCLC showed that carboplatin and paclitaxel could be safely combined with ipilimumab [149]. Interestingly, a “phased regimen” in which immunotherapy began after chemotherapy resulted in substantially increased immune-related progression-free survival in a phase II and phase IIIb/IV study in NSCLC and extensive disease SCLC patients, when compared with chemotherapy alone [162].

Like chemotherapy, localized radiotherapy is currently one of the primary treatments for multiple cancers, and pre-clinical studies have revealed that, in addition to its tumor-debulking properties, radiotherapy modulates the antitumor immune response in a variety of ways. Radiotherapy can lead to the release of tumor antigens and/or DAMPs, such as calreticulin, HMGB1, or ATP, which can activate both the innate and adaptive immune system, and enhance tumor-cell immunogenicity [163]. Clearly, radiotherapy has also been shown to play important roles in enhancing APCs function, overcoming T cell exclusion by reprogramming macrophages, and enhancing T cell effector activity. It is plausible that the immunogenic potential of radiotherapies can be exploited in combination with ICBs to stimulate further immune-mediated tumor destruction. In mice, localized radiotherapy when combined with systemic ICBs resulted in the inhibition of systemic metastases. In humans, several clinical case reports document that the combination of local irradiation and anti-CTLA-4 (iplimumab) in patients with melanoma [164], or NSCLC [165], can result in regression not only of irradiated but also of distant lesions in melanoma and lung cancer patients, also known as the abscopal effect. The contribution of radiotherapy and immune­based therapies to efficacy and resistance in patients with melanoma and in mouse models of melanoma was elegantly delineated: the antitumor activity of combined CTLA4 mAbs and radiotherapy was limited by IFN-γ­driven upregulation of PD-L1 expression, whereas the addition of PD-1 blockade markedly increased therapeutic efficacy, suggesting non-redundant mechanisms induced by the different agents [166]. Limited efficacy of combining radiotherapy and anti-CTLA-4 (iplimumab) was confirmed in a phase I/II study with castration-resistant prostate cancer patients, in which the tumors were first irradiated, followed by anti-CTLA-4 administration two days later in an attempt to maximize antigen presentation [167]. In this study, relatively few severe adverse events were documented and one complete response occurred. However, no differences in median survival were noted in a phase III trial among metastatic castration-resistant prostate cancer patients applying radiotherapy plus iplimumab or placebo, again suggesting that systemic combined effects between radiotherapy and anti-CTLA-4 are sub-optimal [168]. Potential immunosuppressive effects of both chemo- and radiotherapy warrant caution in designing dosing and timing schemes.

Targeted inhibition of different oncogenic signaling pathways has yielded promising therapeutic benefit in some patients with cancer, including KIT inhibitors (imatinib), BRAF inhibitors (vemurafenib and dabrafenib), VEGF inhibitors, PARP inhibitors, and EGFR (erlotinib)- and HER2 (lapatinib)-directed therapies. Unfortunately, diseases tend to relapse and/or develop resistance to these treatments, similarly to what were observed for many conventional therapies [169]. Because targeted antitumor agents can also modulate the tumor immune contexture, the combination of immunotherapy with targeted therapy will probably be a more effective approach for cancer treatment [170–172]. Indeed, increasing evidence from pre-clinical and clinical studies has shown that such combinations would provide better therapeutic outcomes compared with either treatment alone. In one of these studies, the combination of imatinib that can reduce tumor cell expression of IDO and thus, block IDO-mediated immunosuppression of T cell responses, with anti-CTLA-4 mAb resulted in enhanced antitumor response in a mouse model of gastrointestinal tumors [173]. Moreover, imatinib also leads to polarization of TAMs to M2 phenotype, as a result of interaction of macrophages with dying tumor cells, highlighting a possible immune feedback mechanism that could be targeted in order to improve the efficacy of combined immunotherapies [174]. The rationale for the combination of immunotherapy with BRAF inhibitors stems from pre-clinical studies that showed that RAF inhibition resulted in T cell activation and proliferation, consistent with paradoxical activation of the MAPK pathway in BRAF wide-type T cells [175, 176]. Treatment with the BRAF^V600E^ inhibitor vemurafenib appears to improve the antitumor immunity in patients with melanoma, perhaps by enhancing the cross-presentation of antigens from dying tumor cells [171, 177]. However, in a phase I trial, the combination of vemurafenib and anti-CTLA-4 (ipilimumab) led to significant hepatotoxicity, requiring trial discontinuation [178]. Because PD-L1 is elevated on tumor cells following BRAF inhibition, and compared to anti-CTLA-4, anti-PD-1 displays lower toxicity, combining BRAF^V600E^ inhibitors with anti-PD-1 therapy seems promising. Studies using a mouse model of BRAF^V600E^ mutant melanoma showed that PD-1 or PD-L1 blockade combined with BRAF inhibition enhanced the activity of TILs and prolonged survival [179]. Based on these findings, clinical trials evaluating combinations of PD-1 blockade with BRAF inhibitors such as vemurafenib (NCT01656642), and with MEK inhibitors such as trametinib (NCT02224781) are now underway for melanoma. Nevertheless, combinations of the BRAF inhibitor vemurafenib with anti-CTLA-4 or anti-PD-1 mAbs caused various irAEs in patients [178, 180], potentially because of the paradoxical ability of BRAF inhibitors to increase T cell activation through ERK signaling upregulation [175, 176], thus suggesting that the therapeutic index of the combination of targeted therapies with immunotherapy needs to be very carefully assessed.

Tumor vasculature is known to exert immunosuppressive effects via a variety of mechanisms, including decreasing the influx of lymphocytes and DCs in the tumors while increasing the intratumoral frequencies of T_reg_ cells and MDSCs [181]. Thus, anti-angiogenic therapies, a standard-of-care for several malignancies, are correlated with positive immunological changes in neoplastic tissue owing to their ability to normalize aberrant tumor vasculature [182–184]. Therapeutic agents that target VEGF or its receptors, including bevacizumab, sorafenib, and sunitinib, improve tissue perfusion, increase the numbers of intratumoral T_eff_ cells, and reduce accumulation of immunosuppressive T_reg_ cell in RCC [185, 186]. Additionally, treatment with bevacizumab has been associated with enhanced tumor antigen presentation in patients with lung, breast, and colorectal cancers [187]. Several studies reported that the combination of VEGF or VEGF receptor inhibitors with ICB could lead to clinical benefits. In metastatic melanoma, for instance, the combination of bevacizumab and ipilimumab in a phase I study improved immune cell infiltration and augmented efficacy of CTLA-4 blockade [188], and more importantly, humoral immunity to galectin-1 may contribute to the efficacy of anti-VEGF (bevacizumab) and anti-CTLA-4 (ipilimumab) combination therapy [189], offering an additional therapeutic target linking anti-angiogenesis and ICB. Synergistic effects have been reported for combinations of anti-VEGF (bevacizumab and sunitinib) and anti-PD-1/anti-PD-L1 therapies in patients with RCC (reviewed in ref. [135]). Targeting VEGF directly maybe more effective than inhibiting its receptor. Kidney cancers that progress on VEGFR tyrosine kinase inhibitor therapy is correlated with increased VEGF production by the tumor [190]. Many tumor types also secrete VEGF, and elevated VEGF serum levels are a marker of poor prognosis in diverse cancer indications [191]. VEGF can be immunosuppressive because its natural role is to support tissue remodeling and repair. For example, by removing signaling through VEGFR, VEGF blockade can enhance dendritic cell function and subsequent T cell activation [192]. Combining PARP inhibitors, including olaparib, rucaparib, niraparib, veliparib, and talazoparib, with ICBs could be very promising in patients with BRCA-mutated/homologous recombination-deficient (HRD) positive epithelial ovarian cancer (EOC) [193]. In fact, EOCs developing in germline BRCA mutation carriers (gBRCAm, “BRCAness” phenotype) are characterized by a higher mutational load and greater number of neo-antigens that enhance the recruitment of TILs. These cancers often exhibit elevated CD3^+^ and CD8^+^ TILs and increased expression of PD-1 and PD-L1, thus representing a subset of tumors fit for treatment with ICBs alone or in combination with PARP inhibitors or platinum-based chemotherapy [194]. Several clinical trials of combined PARP inhibitors and ICBs are now underway. Four clinical trials (NCT02571725, NCT02734004, NCT02953457, and NCT02484404) are assessing the combination of olapabib and durvalumab and/or tremelimumab as salvage treatment of BRCA1 or BRCA2 mutation carriers with recurrent platinum-sensitive or platinum-resistant or refractory EOC. One trial (NCT02657889) is evaluating niraparib in combination with pembrolizumab in patients with recurrent, platinum-resistant EOC.

In summary, the pre-clinical and clinical studies evaluating combination approaches of ICB with stimulation of antigen release are promising, yet the clinical efficacy is currently limited. Moreover, combination therapies with chemotherapies or targeted therapies are potentially accompanied by severe side effects. Combination approaches with radiotherapy seem to have a more favorable toxicity profile, but to date, local and abscopal antitumor responses are limited. The challenges associated with developing rational combinations of targeted, conventional and immune­based therapies can be organized into three broad, interdependent areas: the requirement for a deeper understanding of the impacts that targeted, conventional and immune­based therapies each have on the patient’s immune system; optimization of efficacy, toxicity, and tolerability through appropriate dosing and sequencing; and a robust approach to prioritizing and resourcing the myriad possibilities for combination therapies.

### Combinations ICBs with CARs

ACT using CAR T cells, which express engineered fusion proteins comprising antigen recognition, signaling, and co-stimulatory domains that could be expressed in CTLs with the purpose of reprogramming the T cells to specifically target tumor cells, has emerged as a very promising approach to combating tumor [195–197]. CAR T cell therapy uses gene transfer technology to engineer a patient’s own T cells to make them stably express CARs, thereby combining the specificity of an antibody with the potent cytotoxic and memory functions of T cells. A prominent example of a clinically successful CAR T cell therapy for the management of B cell malignancies is the second-generation CD19-specific CAR encoding CD28 or 4-1BB signaling moieties (reviewed in ref. [198]). In early-phase clinical trials, CAR T cells targeting CD19 have resulted in sustained complete responses within a population of otherwise refractory patients with B cell malignancies and, more specifically, have shown complete response rates of approximately 90% in patients with relapsed or refractory acute lymphoblastic leukemia [199, 200]. Given this clinical efficacy, pre-clinical development of CAR T cell therapy for multiple tumors has been actively pursued, and the future of the CAR T cell field is extensive and dynamic. However, the effect of CAR T cells has been modest for the treatment of solid tumors due to several factors, including the difficulty in identifying unique tumor-specific antigens, inefficient homing of CAR T cell to tumor locations, their low persistence after infusion, and their functional impairment in the immunosuppressive microenvironment of the solid tumors.

Engineered antitumor T cells need to overcome or to remodel the immunosuppressive microenvironment found in many solid tumors. There are various physical and physiological hurdles faced by T cells in the context of solid tumors. To begin with, T cells must successfully home to the tumor bed, often in the face of mismatches between T cell chemokines and its receptors present in the TME. Moreover, T cells must migrate along an aberrant vasculature that is not conducive to transendothelial migration of T cells, and they can also encounter additional barriers in the stroma, such as suppressive CAFs. Even if T cells are successful in penetrating the tumor bed, they still face a battery of obstacles, including suppressive immune infiltrate comprising T_reg_ cells, MDSCs, TAMs, tumor-associated neutrophils, and immature DCs; suppressive molecules (e.g., TGF-β); suppressive ligands (i.e., PD-L1/L2, VISTA) competition for and/or downregulation of co-stimulatory ligands; and T cell-intrinsic regulatory mechanisms, including PD-1 and CTLA-4 upregulation, and then ultimately exhaustion or anergy [41, 91, 119, 128, 201] (as described above). Finally, the T cells must function in an environment that is acidic, hypoxic, nutritionally depleted, and comprising toxic metabolic by products (i.e., lactic acid) [115, 116]. Thus, even though the engineered T cells can traffic to and precisely recognize tumor cells, they may not be able to effectively attack the tumor when their function is compromised by an immunosuppressive TME. An additional confounding issue is that tumors are heterogeneous in nature, which likely have many different ways to create an immunosuppressive TME, and appropriate countermeasures may need to be tumor specific [41]. An obvious first way to address these problems is by taking advantage of ICBs, such as anti-PD-1, anti-PD-L1, anti-CTLA-4, and others in development, which have been successful in a significant fraction of patients with melanoma or lung cancer.

Irrefutably, ICB therapy can enhance antitumor immune responses by circumventing some of the extrinsic (employed by the tumor) and intrinsic (implicit to T cells) mechanisms that generally facilitate T cell exhaustion. However, this treatment modality may be less efficacious for patients whose pool of antitumor T cells contains a disproportionate mostly terminally differentiated exhausted T cells. It may also be largely ineffective in patients failing to establish immunological antitumor memory following ICB therapy, which may be accompanied by higher rates of tumor relapse. This is particularly evident when considering response to ICBs where the ability to exclude infiltrating immune cells from the TME can make or break an antitumor immune response. Tumors could be classified into several tumor-immune phenotypes, including “inflamed” or “non-inflamed” [202], with more recent reports describing tumors as “immune-deserts,” “immune-excluded,” or “inflamed” [39]. To broaden the scope of treatment options for patients who relapse or fail to exhibit an objective response following ICB therapy, a viable alternative may be to expand the pool of tumor-specific T cells by genetically engineering human T cells. Solid tumor cells also often upregulate immune checkpoint ligands such as PD-L1, which dampens an effector T cell response when engaged with its receptor PD-1, and could lead to inhibition of CAR T cell therapies in the TME. It is possible that some of the ICBs-non-responsive patients may simply lack a population of endogenous T cells that can effectively recognize the tumor (that is “immune-deserts” or “immune-excluded” tumor immune phenotype), even after the checkpoints are inhibited. Thus, combining engineered CAR T cells with ICBs, where PD-1 or PD-L1 antagonists are being co-administered with engineered T cells, makes a great deal of sense, and initial trials appear promising, as evidenced by synergy observed in pre-clinical models. Indeed, ACT plus anti-PD-1, anti-PD-L1, or anti-CTLA-4 synergistically reduced tumor growth in the MC38 carcinoma B16 and B16F10 melanoma mouse models [203, 204], and increased the long-term survival in transgenic Her-2 mice upon ACT of Her-2^+^-specific CAR T cells and anti-PD-1, compared to the monotherapies [205]. Combination therapy enhanced the proliferation of T cells within tumors, and increased their cytotoxic activity, and IFN-γ production, which mediated chemokine elevation (i.e., CXCL10) and further T cell infiltration [203]. Thus, the application of ICB after ACT may result in complete tumor regression in a large population in the clinic. The combination of ACT and anti-CTLA-4 is currently underway in a phase II study in patients with metastatic melanoma (NCT02027935). Because anti-PD-1 mAb and anti-CTLA-4 mAb could promote intratumoral TILs, ICB treatment prior to ACT may increase the number of TILs that can be derived from a tumor biopsy. Furthermore, expanded TILs derived from anti-CTLA-4-treated patients have a less exhausted phenotype, which is associated with improved clinical responses [206]. Applying ICB either before or after ACT is a promising approach, but clinical data are currently lacking.

Various other gene-engineering strategies have been proposed for increasing the activity of CAR T cells in the TME. With respect to the PD-1/PD-L1 checkpoint blockade axis, at least three different approaches have been taken. First, chimeric receptors have been engineered to resist suppression by the checkpoint protein ligand, PD-L1. In this case, the extracellular domain of the checkpoint receptor PD-1 has been fused to intracellular co-stimulatory domains (i.e., CD28), leading to a receptor that will lead to enhanced T cell activity when it engages the normally suppressive PD-L1 signal [207]; second, several recent efforts have been made to use CRISPR genome editing to remove the PD-1 receptor from T cells, rendering them non-responsive to PD-L1-mediated suppression [208, 209]; these types of non-suppressive T cells appear to function well and appear to show enhanced antitumor cell activity. However, it is unclear whether such strategies may lead to increased challenges with control T cell activation. There are many checkpoint molecules that are induced on activated T cells that limit their effector functions, and genetic editing tools permit the efficient disruption of these molecules. However, it is likely that unexpected toxicities will be encountered; third, CAR T cells have been engineered to secrete anti-PD-L1 Abs, all of which have been reported to increase antitumor responses [210]. Others have knocked-down master regulators of T cell activity such as the E3 ubiquitin ligase Cbl-b, showing enhanced antitumor T cell responses [211].

Another general strategy is to equip the engineered T cells with new capabilities, so-called armored CAR T cells constitutively expressing the potent cytokine IL-12 [212, 213], to counteract the suppressive microenvironment. IL-12 is one of the most potent antitumor cytokines, which acts via pleiotropic action on both innate and adaptive immune cells. IL-12 stimulation of T cells results in increased IFN-γ secretion and enhanced expression of the cytolytic proteins granzyme B and perforin, leading to increased cytotoxic capacity, and thus can be a powerful mean to remodel the TME. The advantage of secreted molecules is that they can support not only the T cell that produces them but also endogenous immune cells in the TME. As a final example, co-engineering CAR T cells to constitutively express CD40L showed enhanced T cell proliferation and secretion of pro-inflammatory cytokines. The CD40L^+^ CAR T cells also enhanced the immunogenicity of CD40^+^ tumor cells by the upregulation of co-stimulatory, adhesion, and human leukocyte antigen molecules, as well as the Fas death receptor, and they induced the maturation and secretion of IL-12 by DCs [214]. There are various instances of ICB success in treating different forms of tumor. Additionally, several promising pre-clinical results and emerging models of immunotherapeutic approaches such as CAR T therapy have been reported. Consequently, there is a sound rationale for combining treatment modalities to induce broader antitumor responses. A better understanding of the fundamental mechanisms involved in the development of T cell exhaustion is urgently needed to enhance the therapeutic efficacy of ICB and combination therapies to treat malignancies. This information will also very likely contribute to the optimization of existing and newly emerging CAR T cell strategies.

### Combinations ICBs with epigenetic therapy

Epigenetic dysregulation is a central mechanism in cancer development and progression [215, 216]. Epigenetic events are defined as heritable modifications in gene expression without a change in DNA sequence (i.e., by mutation). Epigenetic alterations also affect chromatin structure by post-translational modifications of histone tails and remodeling of nucleosome without changes to the underlying nucleotide sequence [216–218].

Cancer epigenetic silencing is often characterized by EZH2-mediated histone modifications and DNMT-mediated DNA methylation, and is a common strategy used by tumor cells to escape immune surveillance, by downregulating of tumor-associated antigens or molecules that are required for processing and presentation of these antigens and thereby, or interfering with recognition of tumor cells by the immune system. The use of epigenetic drugs in sensitizing immunotherapeutic responses via their ability to modulate tumor cell-immune interaction and reverse crucial elements of immune evasion has been justified by growing evidence. Since some epigenetic regulators have shown a potent immunomodulatory activity, their combination with ICBs could represent a promising therapeutic strategy. Many pre-clinical studies and clinical investigations have been conducted using different classes of HDACi in combination with immunotherapeutics. HDACis appear to be able to improve the in vivo therapy efficacy, and even if other pre-clinical data are needed to assess the efficacy and toxicity of these drugs alone or in combination with other chemotherapeutics and immunotherapy strategies. In an important study that paved the way for combined epigenetic and immunotherapy, dual epigenetic therapy with azacytidine and entinostat (a class I HDACi) failed to display significant antitumor responses in patients with advanced lung cancer. However, in this study, a small number of patients with advanced NSCLC who progressed after receiving low-dose epigenetic therapy entered a trial for ICB therapy with an anti-PD-1 checkpoint inhibitor (nivolumab) [219, 220]. Tellingly, five of six (83%) patients survived 6 months post-treatment without cancer progression, an unexpected outcome for immunotherapy in NSCLC, sparking significant interest in the potential of combining epigenetic and immunotherapy in not only NSCLC but also in other tumor types (i.e., melanoma, prostate cancer, and colon cancer) [221, 222]. Due to the explosion of interest in cancer immunotherapy, there is a plethora of new research on epigenetic drugs used in combination with different immunotherapies, including ACT, immunostimulatory mAbs, cytokine-based therapy, and vaccination strategies; thus, we focuses specifically on combinations with ICBs as the following.

Epigenetic modulators can enhance responses to ICBs by several mechanisms such as increasing expression of checkpoint inhibitors on tumor cells, induction of chemokine expression on T cells, and reduction of suppressive cell populations in the TME. Several studies have shown that increased expression of checkpoint inhibitors on tumor cells following epigenetic treatment enhances responses to ICB therapy [220, 223], for instance, a study from Woods et al. showed that treatment with HDACis in melanoma-bearing mice elevated PD-L1 and PD-L2 in tumor cells as a result of increased histone acetylation, and combined HDACi and anti-PD-1 therapy slowed tumor progression and increased survival compared to single-agent therapy [223]. Additionally, epigenetic modulators have been shown to increase T cell infiltration into the TME and augment responses to ICBs through the removal of epigenetic marks suppressing chemokine expression in cancer [222, 224–226]. It was recently shown that tumor cells can escape immune surveillance through the epigenetic repression of chemokines important for immune cells infiltration of the TME. Tumor production of the T helper 1 (Th1)-type chemokines CXCL9 and CXCL10 is epigenetically repressed by EZH2-mediated H3K27me3 and DNMT1-mediated DNA methylation in EOC [222]. Epigenetic modulation using a DNMTi was able to induce chemokine expression and result in increased tumor infiltration of T cells and an improved therapeutic response to anti-PD-L1 compared to single therapy alone. Moreover, HDACis have also been shown to upregulate T cell chemokine expression and TME infiltration and enhance responses to PD-1 therapy in lung cancer [226]. Similar results have also been shown with ICBs targeting anti-CTLA-4 [225]. In addition to increasing T cell infiltration, HDACis could also reduce suppressive cell populations such as MDSCs to augment ICB therapy. A recent study examined the effects of two key checkpoint inhibitors, anti-PD-1 and anti-CTLA-4, in conjunction with two epigenetic modulating drugs (5-azacytidine and entinostat) in colorectal- or metastatic breast tumor-bearing mice, resulting in primary tumor eradication in about 90% mice with colorectal cancers and all mice with metastatic breast cancer [227]. Moreover, metastasis did not develop in the metastatic breast cancer model following combination treatment compared to single therapies alone. Further functional studies showed that these epigenetic drugs acted by blocking the suppressive activity of tumor-infiltrating G-MDSCs against T cell killing. However, while entinostat was shown to reduce G-MDSC viability, the specific mechanisms underlying the targeted suppression of G-MDSCs by epigenetic drugs were not examined. This study also highlighted the potential for using combined epigenetic and immunotherapy against minimally immunogenic cancers, which are very difficult to eradicate, in either mice or humans.

Immunotherapy offers many distinct advantages over conventional cancer treatment regimens, including the potential to be applied globally to different tumor subtypes and to elicit specific and durable responses by immunological memory. The ability of epigenetic drugs to specifically prime epithelial cancer cells for host immune responses holds significant promise for future immunotherapy in patients with tumor. Indeed, a number of epigenetic and immunotherapeutic drug regimens have already been used or are under intense investigation in different tumor models (i.e., colon, breast, and melanoma) (reviewed in ref. [228]). Several clinical trial studies have a run-in phase with sequential single agents and then a combination phase. Thirty patients will be enrolled in 2 expansion cohorts: 15 anti-PD-1-naïve patients and 15 anti-PD-1-resistant patients (NCT02619253). Focusing on DNMTi, 5-azacytidine or entinostat will be orally administered to metastatic NSCLC patients together with the anti-PD-1 mAb (nivolumab, NCT01928576). In a phase I study, the safety of a combination between orally administered pembrolizumab and 5-azacytidine will be evaluated (NCT02546986). Likewise, in a phase II study, 60 patients with NSCLC will be enrolled to evaluate the efficacy of decitabine plus nivolumab treatment versus nivolumab alone (NCT02664181). Even if the function of HDACis and DNMTis in immune priming of tumor cells has been explored reasonably thoroughly, epigenetic drugs, such as decitabine, can have pharmacological limitations, such as a short half-life, sensitivity to inactivation by cytidine deaminase in vivo, and pronounced hematopoietic toxicity, all of which might impede its use in combination regimens. To address these concerns, other epigenetic drugs are currently in development. Pre-clinical experience of SG110, a dinucleotide of decitabine, has shown that it is more convenient and tolerated, achieving biologically relevant hypomethylating effects at lower and less myelosuppressive doses than decitabine while displaying immunomodulatory activity [228]. More importantly, a trial combining the DNMTi SGI­100 (also known as guadecitabine) with CTLA4 blockade (ipilimumab) is recruiting patients with metastatic melanoma (NCT02608437). Additionally, there are several other classes of epigenetic drugs, including histone methyltransferases inhibitors (i.e., GSK126) [222], bromodomain inhibitors (i.e., JQ1) [229], and histone demethylase inhibitors (i.e., INCB059872) [230], which have recently been reported to increase immune signaling in tumor cells. In addition, several epigenetic drugs are in clinical trials or under investigation in cancer and other human diseases that may useful in combination with immunotherapies.

### Fine-turning ICB therapy: optimizing the gut microbiome

The equilibrium linking the intestinal microbiota, the intestinal epithelium, and the host immune system determines host health and homeostasis. A number of studies have led to an understanding that gut microbiota and antitumor interventions profoundly affect each other [231]. It was recently shown that the gut microbiota, intestinal epithelial cells, and host immunity interactions influence carcinogenesis and the efficacy of antitumor treatment and gut microbiota play a role in immune system development and can affect the occurrence of autoimmunity. Both chemo- and radiotherapy result in gastrointestinal mucositis in a significant proportion of patients with tumor and can have direct or indirect (e.g., immune-mediated) cytotoxic effects on intestinal bacteria, thus culminating in dysbiosis [232]. Alternatively, or additionally, the gut microbiota could influence both therapeutic and adverse effects of antitumor interventions either by pharmacodynamic or immunological mechanisms [231]. A plethora of initial breakthroughs linking the gut microbiota to the immune-mediated efficacies of antitumor therapies highlighted the importance of an intact commensal microbiota in cancer therapy and suggested the possibility of establishing a favorable microbiota in patients with ineffective pre-existing enteric microbial microflora that might be correlated with a poor prognosis.

Recent evidence provides further insight into the therapeutic effects of CTLA-4 blockade, revealing that the immunostimulatory and antitumor effects of this ICB depend on distinct *Bacteroides* species of the gut microbiota [233]. CTLA-4 mAb lost its therapeutic efficacy against established melanomas, colon cancers, and sarcomas in mice that were reared under germ-free conditions or that had been raised in specific pathogen-free environment and then treated with multiple broad-spectrum antibiotics to sterilize the gut. This defect was overcome by gavage with *Bacteroides fragilis*, by immunization with *B*. *fragilis* polysaccharides, or by adoptive transfer of *B*. *fragilis*-specific T cells, suggesting a therapy-relevant cross-reactivity between microbial and tumor antigens recognized by the same TCR. Accordingly, both in mice and in patients, T cell responses specific for distinct *Bacteroides* species (*B*. *fragilis* and *B*. *theraiotaomicron*) were correlated with the administration and efficacy of CTLA-4 blockade. Furthermore, fecal microbial transplantation of feces harvested from each of these patient clusters into germ-free tumor-bearing mice highlighted that the microbial composition of cluster C, rich in immunogenic *Bacteroides* spp. (mainly contributing to the niching of *B*. *fragilis*), could restore anti-CTLA-4 mAb efficacy, whereas cluster B enriched with tolerogenic *Bacteroides* species mediated complete resistance to the mAb. A parallel study to that above has shown a role for distinct components of the gut microbiota, especially *Bifidobacterium*, in promoting natural antitumor immune responses [234]. Sivan et al. compared the antitumor CTL responses in genetically similar C57BL/6 tumor bearers derived from two different mouse facilities, the Jackson Laboratory (JAX) and Taconic Farms (TAC), to have differing commensal microbes. JAX and TAC mice exhibited significant differences in the growth kinetics of subcutaneously implanted melanomas; more aggressive tumors in TAC mice were attributable to lower tumor-specific T cell responses elicited in draining lymph nodes and poor intratumoral accumulation of tumor-antigen-specific CD8^+^ T cells. The aggressive neoplastic growth in TAC mice could be reduced to the rates seen in JAX mice after either JAX fecal transplantation or cohousing between the mice. Notably, *Bifidobacterium* was identified as associated with the enhanced tumor control. Hence, oral feeding of TAC mice with *Bifidobacterium* or cohousing of TAC and JAX mice restored CTL responses and allowed the host to control tumor progression by activating the processing and presentation machinery of intratumoral immune cells. More importantly, the microbiome also effects the therapeutic efficacy of PD-L1 blockade. Injection of a blocking antibody against PD-L1 was much more efficient in reducing the growth of melanomas in mice containing a high abundance of *Bifidobacterium* in their gut than in mice lacking this genus. *Bifidobacterium*-induced TIL enrichment of the TME also allowed for enhanced antitumor effects mediated by anti-PD-L1 mAb immunotherapy. DCs purified from mice that had been treated with *Bifidobacterium* were particularly active in presenting a melanoma-driven peptide antigen to T cells for stimulation of their proliferation and IFN-γ production, suggesting that *Bifidobacterium* improves the antitumor immune response by an effect on DCs. Hence, the mechanistic bases of the microbial contribution to the mode of action of distinct checkpoint blockers share common features but might also somewhat differ. While both studies describe the gut microbiota-dependent intratumoral maturation of DCs, the first study (on anti-CTLA4) suggests a role for cross-reactive T cell epitopes present on bacteria and cancer, the latter (on anti-PD-L1) postulates an effect on innate immunity leading to a gut microbiota-dependent resetting of antigen presenting cell functions. In addition to pre-clinical mouse models, two recent papers reveal that gut microbiome can also influence the efficacy of PD-1-based immunotherapy against epithelial tumors and melanoma in patients [235, 236]. In one article, Zitvogel and collaborators show that fecal microbiota transplantation from cancer patients who responded to ICB into germ-free or antibiotics-treated mice alleviates the antitumor effects of PD-1 blockade [235]. Notably, oral supplementation with *Akkermansia muciniphila* post-fecal microbiota transplantation with non-responder faces restored the efficacy of PD-1 blockade in an IL-12-dependent manner by increasing the recruitment of CCR9^+^CXCR3^+^CD4^+^ T lymphocytes into tumor beds. Whereas in another paper, Wargo and colleagues demonstrate that significant differences were observed in the diversity and composition of the patient gut microbiome of responders versus non-responders, and analysis of patient fecal microbiome samples showed significantly higher alpha diversity and relative abundance of Ruminococcaceae bacteria in responding patients [236]. Tellingly, immune profiling suggested enhanced systemic and antitumor immunity in responding patients with a favorable gut microbiome, as well as in germ-free mice receiving fecal transplants from responding patients.

The aforementioned examples illustrate that antitumor therapies aiming at restricting immunosurveillance are profoundly influenced in their efficacy by the gut microflora that can act at distance, on a range of a priori sterile tumors. The utility of ICBs comes at the price of gastrointestinal and hepatic complications. Severe gastrointestinal toxicity, particularly colitis, frequently occurs in patients upon immunotherapy, additionally, hepatitis, diarrhea, and enterocolitis are characteristic side effects of ICBs that result from a complex interplay of host genetics, immune responses, environment, and the microbiota [237, 238]. Notably, immune responses following CTLA-4 blockade with ipilimumab not only execute their program systemically in secondary lymphoid organs contributing to reinstating tumor immunosurveillance but also are directed at sites where microbiota are abundant [233]. Indeed, patients treated with ipilimumab develop anti-microbiota antibodies that appear to be correlated with irAEs, thus, gut microbiota might be implicated in irAEs, and the eagerly anticipated breakthrough of one day being able to uncouple efficacy from toxicity could stem from a better understanding of the disequilibrium between commensals and pathobionts. Tellingly, the recent evidence that inter-individual differences in intestinal microflora are a source of the heterogeneity in immunotherapeutic and toxicity indicates that it might be possible to improve the therapeutic index of ICBs with “adjunctive oncomicrobiotics,” potentially including live immunogenic commensals, derivatives of such commensals, and/or perhaps antibiotics that selectively target immunosuppressive microbes. Such improvements in efficacy correspond to directing increased numbers of TILs of the right functionality have been indicated by several studies [233, 234, 239, 240]. Theoretically, TIL infiltration depends on the quality of the priming of naïve T cells, which is determined by the presence of DCs in the proximity of dying or apoptotic tumor cells, by existing neoantigens or high mutational load allowing high-avidity TCR engagement and proliferation [241], and by an appropriate “immunogenic milieu” which DAMPs can be released [153]. Besides to these aspects, another input to the immunogenic milieu responsible for quality TILs, or the reinstating of defective antitumor T cell responses, is derived from immune sensing of the bacterial species present at that particular time in the microflora.

A plethora of tumor- and host-related factors combine in diverse ways to define the heterogeneity in clinical responses to ICB antitumor therapy. A variety of adjunctive therapeutics are being combined with ICBs to reduce this heterogeneity by improving the probability, duration, and potency of clinical activity; many of these have seen success, albeit often in parallel with increased toxicities. A new breakthrough in this arena, which could be feasible beside multi- (or mono-) therapeutic strategies, is manipulation of the gut microbiota. Just as the microbiome is modified during cancer, possibly supporting oncogenesis, we have the chance to manipulate the microbiome toward a status that promotes immune-mediated tumor control. However, the exact microbial constitution of this status for a given ICB (or other immunotherapy) and the particular approaches clinicians should use to manipulate the microbiome remain to be determined.

## Conclusions

Studies of the interplay between immune activation and suppression have shown an important role for immune checkpoints in the pathogenesis of malignancies. An improved understanding of the immune response to tumor, as well as patient selection and biomarker development, has increased the number of patients who benefit from checkpoint blockers. However, the benefit, to date, has been limited to a minority of patients with certain tumor types. The goal of combination immunotherapies is to produce a durable antitumor response in patients who would not benefit from monotherapy. Bringing clinical benefit to most patients requires a complete understanding of the mechanisms that would lead to an effective antitumor response and the different tumor- and host-related factors that could combine in diverse ways to define the heterogeneity in clinical responses to ICB therapy and would result in primary, adaptive, and acquired resistance to immunotherapy. Dynamic immunologic studies along with genetics and epigenetics in the human TME will guide the development of different combination therapies and generate novel insight into how the human immune system responds to and is shaped by a variety of tumor types.

## Abbreviations

ACT: Adoptive T cell therapy
APC: Antigen-presenting cells
CIC: Cancer-immunity cycle
CTLA-4: Cytotoxic T-lymphocyte protein 4
CTLs: Cytotoxic T lymphocytes
DCs: Dendritic cells
EOC: Epithelial ovarian cancer
HL: Hodgkin’s lymphoma
HRD: Homologous recombination deficient
ICBs: Immune checkpoint blockades
ICD: Immunogenic cell death
IDO: Indolaimine-2,3-deoxygenase
IRF1: Interferon regulatory factor 1
KIRs: Killer inhibitory receptors
MDSCs: Marrow-derived suppressor cells
MHC: Major histocompatibility complex
MSI: Microsatellite instability
NK: Natural killer
NSCLC: Non-small-cell lung cancer
ORR: Overall response rate
PD-1: Programmed cell death protein 1
RCC: Renal-cell carcinoma
TAMs: Tumor-associated macrophages
TCR: T cell receptor
T_eff_: Effector T cells
TILs: Tumor-infiltrating T cells
TME: Tumor microenviroment
T_regs_: Regulatory T cells
